# Associations of colonic transit with the gut microbiota and short-chain fatty acids in health and irritable bowel syndrome

**DOI:** 10.3389/fnut.2026.1953842

**Published:** 2026-10-08

**Authors:** Xiang Gao, Qunfeng Dong, Andrea S. Shin

**Affiliations:** 1 Department of Medicine, Stritch School of Medicine, Loyola University Chicago, Maywood, IL, United States; 2 Center for Biomedical Informatics, Stritch School of Medicine, Loyola University Chicago, Maywood, IL, United States; 3 Vatche and Tamar Manoukian Division of Digestive Diseases, University of California, Los Angeles, Los Angeles, CA, United States; 4 Goodman-Luskin Microbiome Center, David Geffen School of Medicine at the University of California, Los Angeles, Los Angeles, CA, United States; 5 Walter and Shirley Wang Center for Integrative Digestive Health, University of California, Los Angeles, Los Angeles, CA, United States

**Keywords:** colonic transit, gut microbiome, irritable bowel syndrome, mediation analysis, short-chain fatty acids

## Abstract

Gut bacteria are known to influence short-chain fatty acid (SCFA) levels through direct metabolic activity. Colonic transit also affects substrate contact time, fermentation duration, and SCFA absorption. But it is unclear whether some bacteria influence SCFA levels indirectly, by altering transit, rather than only directly, and whether this differs across health and irritable bowel syndrome (IBS) subtypes. Using data from 56 participants [17 healthy volunteers (HV), 15 IBS-C, 24 IBS-D] with concurrent stool metagenomes, stool SCFA, and segmental colonic transit measurements, we applied mediation analysis to test whether each bacterial taxon's association with SCFA levels may be transit-mediated statistically. Three taxa showed a potential transit-mediated association with at least one SCFA: *Faecalibacterium prausnitzii* in HV, *Bacteroides thetaiotaomicron* in IBS-C, and *Phascolarctobacterium succinatutens* in IBS-D. For *F. prausnitzii*, the transit-mediated pathway was the only detectable association with SCFA. The remaining two taxa, *B. thetaiotaomicron* and *P. succinatutens*, additionally showed a direct, transit-independent effect alongside the transit-mediated one. Separately, an analysis of direct, non-mediated effects identified 15 additional taxa (5 in HV, 2 in IBS-C, and 8 in IBS-D) whose associations with SCFA levels were not explained by colonic transit. Together, these findings suggest that colonic transit may potentially mediate specific associations between gut bacteria and SCFA levels, in addition to the direct metabolic effects of the bacteria. However, because the present study is cross-sectional, these findings should be interpreted as statistical associations rather than established biological or directional relationships. In addition, this was an exploratory *post-hoc* analysis of a single relatively small cohort, and independent validation will be required before the biological or clinical significance of these findings can be established.

## 1 Introduction

Irritable bowel syndrome (IBS) is a prevalent disorder of gut-brain interaction, affecting an estimated 5%−10% of the global population (1–3). It is characterized by recurrent abdominal pain and altered bowel habits and classified into subtypes based on predominant stool form, including IBS with constipation (IBS-C), IBS with diarrhea (IBS-D), IBS with mixed bowel habits, and IBS with unclassified bowel habits (4). Among the pathophysiological mechanisms implicated in IBS, disturbances in the gut microbiome and altered microbial metabolite production have each received considerable attention due to their established role in modulating critical aspects of gastrointestinal (GI) physiology. A systematic review and meta-analysis found that, despite substantial heterogeneity in methods and populations, IBS is consistently associated with altered gut microbial composition relative to health, though no single taxonomic signature has been reproducible across all studies (5).

Short-chain fatty acids (SCFA), including acetate, propionate, and butyrate, are key products of anaerobic microbial fermentation that regulate intestinal homeostasis and immune cell differentiation (6, 7). Taxon-specific fermentation capacity and substrate preference shape the quantity and composition of luminal SCFA, such that bacterial community composition directly influences SCFA profiles (6). Colonic transit time separately affects SCFA levels. Longer intestinal transit time has been associated with lower fecal SCFA and plasma acetate in healthy adults (8). Experimentally accelerating intestinal transit in healthy volunteers increases fecal butyrate concentration, while slowing it decreases fecal butyrate (9). Faster colonic transit has also been associated with higher fecal SCFA concentrations across a cohort including both healthy volunteers and patients with IBS (10), suggesting that transit may substantially influence microbiota-SCFA relationships in IBS. This relationship may be particularly relevant to understanding the role of the gut microbiome in IBS in which a subset of individuals may exhibit altered intestinal transit. However, whether these observed associations reflect altered colonic absorption, altered fermentation dynamics, or both remains unresolved. Although SCFA are produced by bacterial fermentation of luminal substrates, gut bacteria are, in turn, capable of directly modulating intestinal transit. Proposed mechanisms include the production of neuroactive metabolites, direct interaction with enteric neurons, and effects on mucosal serotonin signaling (11). Whether associations between specific taxa and SCFA are explained by microbial effects on transit, rather than direct metabolic activity alone remains unclear and has not been investigated in healthy individuals or in IBS subtypes.

We previously reported that microbiota-SCFA associations differ across IBS subtypes, using a statistical approach that conditioned on transit time to isolate microbe-SCFA associations independent of transit (12). While appropriate for that purpose, conditioning on transit removes its contribution to the microbiota-SCFA relationship entirely and therefore cannot determine whether bacteria may influence SCFA indirectly by modulating motility transit. Quantifying this indirect pathway is conceptually distinct from removing it, and is important because it identifies which microbe-SCFA associations are mediated transit and may have direct implications for clinical care by informing whether motility-targeted, dietary, or microbiome-targeted therapies are more appropriate for a given taxon-SCFA association.

Mediation analysis provides a formal statistical framework for decomposing the total association between an exposure (bacterial abundance) and an outcome (SCFA concentration) into a direct component and an indirect component operating through a specified mediator (transit time) (13, 14). This framework can address a question that conditioning-based approaches cannot: do gut bacteria show a statistical association with SCFA levels that is consistent with a direct pathway, an indirect pathway operating through colonic transit, or both simultaneously and even in opposing directions? In this study, we performed a *post-hoc* analysis using data from our prior cohort (12), applying mediation analysis separately within HV, IBS-C, and IBS-D to test whether colonic transit time may have the potential to statistically mediate the association between gut bacteria and stool SCFA concentrations.

## 2 Materials and methods

### 2.1 Literature search for prior work

To assess whether mediation analysis of colonic transit as a mediator between gut bacteria and SCFA levels had previously been reported, we searched PubMed (last done on July 26, 2026) using the query: (“Gastrointestinal Transit”[MeSH] OR “colonic transit” OR “gut transit” OR “bowel transit” OR “intestinal transit”) AND (“Gastrointestinal Microbiome”[MeSH] OR “gut microbiota” OR “gut microbiome” OR “gut bacteria”) AND (“Fatty Acids, Volatile”[MeSH] OR “short-chain fatty acid^*^” OR SCFA OR SCFAs OR acetate OR propionate OR butyrate) AND (“mediation analysis” OR “causal mediation” OR mediator OR “indirect effect” OR ACME). This search returned 22 results. None applied formal statistical mediation analysis with gut bacteria as the exposure, colonic transit as the mediator, and SCFA as the outcome. The closest prior study, from our own group, used partial canonical correspondence analysis conditioned on transit time to isolate microbe-SCFA associations independent of transit, rather than quantifying transit's mediating contribution (12).

### 2.2 Study population and design

We conducted a *post-hoc* analysis of data from our previously published cohort of adults with IBS according to Rome IV criteria and healthy volunteers (HV) with no prior history of GI diseases or disorders (12). The Indiana University Institutional Review Board approved this prior study, and the protocol was registered with ClinicalTrials.gov (NCT02981888). The analytic cohort for this *post-hoc* analysis comprised 56 participants (17 HV, 15 IBS-C, 24 IBS-D) with concurrent stool metagenomic, stool SCFA, and colonic transit data available (Supplementary Table S1).

### 2.3 Fecal sample collection

As previously described (12), stool specimens were collected over 48 h during the last 2 days of a standardized 4-day, 100 g fat diet, consistent with clinically validated methods for identifying bile acid malabsorption. Specimens were refrigerated during the collection period, returned to the research team on ice, and stored at −80 °C until processing.

### 2.4 Stool SCFA and colonic transit measurements

As previously described (12), stool SCFA (acetate, propionate, butyrate) were measured by liquid chromatography-mass spectrometry, and overall and segmental (right colon, transverse colon, left colon) colonic transit times were measured in days using a validated radiopaque marker method.

### 2.5 DNA extraction, library preparation, and sequencing

As previously described (12), genomic DNA was isolated from stool using the QIAamp PowerFecal DNA kit (QIAGEN Inc., Germantown, MD, USA), and DNA quality and concentration were assessed by Qubit fluorometer. Purified libraries were prepared using Nextera XT (Illumina) and sequenced as paired-end 2 × 150 bp reads on a NovaSeq v1.5 SP (Illumina, San Diego, CA, USA), targeting a sequencing depth of approximately 40 million read pairs per sample.

### 2.6 Metagenomic data processing

Raw sequencing reads were quality filtered. Across the analytic cohort (*n* = 56), total sequencing depth averaged 48.4 ± 16.5 million reads per sample (mean ± SD), of which an average of 22.4% ± 11.8% (mean ± SD) were of human origin and were removed. Species-level taxonomic classification of the remaining, non-human reads was performed with MetaPhlAn3 (15) using default parameters, yielding relative-abundance profiles for 463 species-level taxa.

From these 463 species-level taxa, a candidate set of 105 species-level taxa was selected for the current analysis using a prevalence- and abundance-based filtering approach consistent with our prior study of this cohort, which retained taxa present at a relative abundance of at least 0.1% in at least two specimens within at least one clinical group (HV, IBS-C, or IBS-D) (12).

### 2.7 Taxon abundance estimation: two independent normalization methods

Because compositional microbiome data require a normalization or bias-correction step whose choice can in principle influence results, we quantified taxon abundance using two independent, methodologically unrelated approaches and required that findings be consistent across both before highlighting them as a primary result.

#### 2.7.1 ANCOM-BC2

We applied ANCOM-BC2 (16, 17) to the classified species-level read counts for the full cohort (*N* = 56), using clinical group (HV, IBS-C, IBS-D) as the fixed effect and a prevalence filter of 10%, to produce a bias-corrected log-abundance estimate for a total of 94 taxa in each sample. In addition, a within-group sensitivity re-analysis, in which ANCOM-BC2 was instead applied separately within each clinical group (HV, IBS-C, IBS-D) rather than to the pooled cohort, confirmed nearly identical bias-corrected abundance estimates (median Pearson correlation *r* = 0.982 across all 94 taxa) (Supplementary Table S2).

#### 2.7.2 Centered log-ratio (CLR) transformation

As an independent sensitivity analysis using an unrelated compositional-data method, taxon relative abundances were transformed by centered log-ratio using the microViz R package (18) with its default setting. Specifically, for each sample, each taxon's proportion was log-transformed after addition of a pseudocount, defined as half of the minimum non-zero relative abundance observed in the 94-taxon matched dataset (4.62 × 10^−7^ based on *Alistipes shahii*) (19). Each sample was then centered by subtracting the mean log-proportion across all taxa in that sample. To ensure that the two abundance-estimation methods tested identical taxa, CLR was computed using the same 94 taxa retained by ANCOM-BC2's prevalence filter, with the CLR geometric-mean denominator restricted accordingly to these 94 taxa.

Raw read counts for the 94 analyzed taxa, along with the CLR-transformed and ANCOM-BC2 bias-corrected abundance values used throughout, are provided in Supplementary Table S1.

### 2.8 Statistical analysis

The causal structure assumed by the subsequent mediation analysis is shown as a directed acyclic graph in Supplementary Figure S4, together with the measured (age, sex, BMI) and unmeasured (medication use, stool characteristics, and other physiology) covariates relevant to this structure. All analyses were performed in R (v4.5.0; https://cran.r-project.org/bin/windows/base/old/4.5.0/). Because mediation and path models were fit separately within each clinical group, SCFA concentrations, transit times, and bacterial abundances (ANCOM-BC2 bias-corrected log abundances or CLR-transformed values) were z-scored within each clinical group prior to mediation and path modeling, so that path coefficients reflect standardized within-group associations rather than between-group differences in mean transit, SCFA, or bacterial abundance. The analysis proceeded in two stages: Stage 1 screened each taxon in a separate single-mediator model for every combination of transit segment and SCFA to identify candidate transit-mediated associations within each clinical group; Stage 2 then modeled the taxa surviving this screen in a joint path model of all three SCFAs. This two-stage strategy was adopted for two reasons. First, given the large number of taxa screened, Stage 2 only needs to be applied, with multiple-testing correction, to the small number of candidates identified by Stage 1. Second, because acetate, propionate, and butyrate are correlated with one another, jointly modeling them as correlated outcomes in Stage 2 can recover statistical power that Stage 1's single-outcome screen loses. The full taxon flow through this two-stage pipeline, from the initial candidate set to the final potential findings, is shown in Supplementary Figure S1.

#### 2.8.1 Stage 1: single-outcome mediation analysis

For each clinical group and each abundance-estimation method (ANCOM-BC2; CLR), we tested whether each of four transit measures (total, right colon, transverse colon, left colon) mediated the relationship between bacterial abundance and each of three SCFAs (acetate, propionate, butyrate). This yielded 1,128 mediation tests per group under each abundance-estimation method (94 taxa × 4 transit segments × 3 SCFAs). We used the mediation R package (13) with the mediator model (Transit ~ Taxon) and outcome model (SCFA ~ Taxon + Transit).

Clinical group was not included as a covariate because analyses were conducted within each group. The average causal mediation effect (ACME, the indirect effect through transit), average direct effect (ADE, the transit-independent effect), total effect (the direct and indirect effects combined, equivalent to the taxon's overall, unadjusted association with the SCFA), and proportion mediated were estimated with 1,000 bootstrap replicates and percentile confidence intervals. Because this stage screens a large number of taxon-transit-SCFA combinations, the ADE *p*-values were corrected for multiple testing using the Benjamini-Hochberg false discovery rate (FDR) method to identify significant direct effects. For the ACME (indirect effect), the nominal, uncorrected *p*-value for this single-outcome model was used at this stage as a screening threshold for entry into Stage 2′s multiple-outcome path models (Section 2.8.2).

#### 2.8.2 Stage 2: multiple-outcome path models

To extend the single-outcome mediation screen, we evaluated, using path models that jointly modeled all three SCFAs as correlated outcomes, every taxon reaching a nominal (uncorrected) ACME p < 0.05 for at least one taxon-transit-SCFA combination under both abundance-estimation methods. The uncorrected ACME *p*-values were used because a screening stage is generally expected to use a more permissive threshold. For each qualifying taxon, we fit a separate single-mediator path model for every transit segment reaching ACME *p* < 0.05 in Stage 1. Each single-mediator path model was fit using lavaan (20) with the following structure: one a-path (taxon to transit), three b-paths (transit to each SCFA, controlling for taxon), three c'-paths (direct taxon to each SCFA), and three SCFA residual covariances, totaling 10 estimated parameters. Specific indirect effects (a × b) were computed for each SCFA, which represents the portion of the taxon's association with the SCFA that is explained by its association with transit: the taxon-transit path (a-path) multiplied by the transit-SCFA path (b-path). Its statistical significance was assessed by recomputing this product at each bootstrap resample of the data, yielding an empirical sampling distribution, confidence interval, and *p*-value for the indirect effect itself. Models were estimated with bootstrap standard errors (1,000 replicates) and percentile confidence intervals.

We did not apply FDR correction to the results of Stage 2 because the appropriate multiple-testing family is less straightforward: at Stage 2, the three SCFA outcomes examined for each taxon are correlated, and there is no single generally accepted way to define the unit of multiplicity correction. Instead, we conducted two additional diagnostics to further assess the consistency of the Stage 2 findings. First, a leave-one-out sensitivity analysis refit the Stage 2 model to assess whether the sign and magnitude of the specific indirect effects were driven by one or a small number of influential observations. Second, because percentile bootstrap confidence intervals rely on asymptotic assumptions that may be imprecise at our modest sample sizes, we additionally conducted a permutation test. Within each clinical group, taxon abundance values were permuted across participants while transit time and all three SCFA measurements were held fixed, the Stage 2 model was refit to the permuted dataset, and this was repeated 2,000 times per group to generate an empirical null distribution and *p*-value for each indirect effect, defined as the proportion of permuted effects at least as large in magnitude as the observed effect. A Stage 2 indirect effect was classified as consistent only if it reached *p* < 0.05 by both the percentile-bootstrap and the permutation test, under both ANCOM-BC2 and CLR normalization.

## 3 Results

### 3.1 Study population and transit-time characteristics

The analytic cohort comprised 56 participants: 17 healthy volunteers (HV), 15 with IBS-C, and 24 with IBS-D, drawn from our previously published cohort (12). For this cohort, age (mean ± SD: HV 31.7 ± 13.6, IBS-C 34.3 ± 10.5, IBS-D 37.5 ± 13.4 years) and BMI (mean ± SD: HV 26.3 ± 5.9, IBS-C 25.3 ± 5.2, IBS-D 28.8 ± 5.7 kg/m^2^) did not differ significantly between groups (ANOVA: age *p* = 0.36, BMI *p* = 0.15), nor did sex (women: HV 71%, IBS-C 87%, IBS-D 71%; Fisher's exact test, *p* = 0.50) (12).

Median transit time was numerically slower in IBS-C than in IBS-D for three of the four measures (total, transverse colon, and left colon transit; Table 1), and equal between groups for right colon transit, consistent with the constipation- and diarrhea-predominant nature of these subtypes. Because all four transit measures were right-skewed and departed significantly from normality (Shapiro-Wilk *p* < 0.02 for each), group differences were assessed with the Kruskal-Wallis test. None of the four measures differed significantly between groups (Kruskal-Wallis *p* = 0.11 for total, 0.75 for right, 0.49 for transverse, and 0.07 for left colon transit), although the result for left colon transit was borderline given this modest sample size.

**Table 1:** Colonic transit-time characteristics of the analytic cohort.

Transit-time measure	HV (*n* = 17)	IBS-C (*n* = 15)	IBS-D (*n* = 24)
Total colonic transit time	1.40 [0.90, 2.50]	1.50 [0.95, 2.05]	0.95 [0.57, 1.52]
Right colon transit time	0.60 [0.30, 0.60]	0.40 [0.20, 0.75]	0.40 [0.20, 0.60]
Transverse colon transit time	0.10 [0.00, 0.40]	0.30 [0.00, 0.80]	0.10 [0.00, 0.35]
Left colon transit time	0.50 [0.30, 1.90]	0.50 [0.15, 1.05]	0.30 [0.10, 0.53]

Values are presented as median [IQR] and are expressed in days. IQR, interquartile range.

Total colonic transit time was highly correlated with left colon transit (*r* = 0.89, *p* < 0.001), but right, transverse, and left colon transit times were largely independent of each other (*r* = 0.18–0.19, all *p* > 0.15).

### 3.2 Transit-mediated associations by group

Stage 1 screened each taxon individually for a direct (ADE) or indirect (ACME) association with each SCFA; taxa with a significant indirect signal then entered Stage 2, which jointly modeled all three SCFAs in a single path model.

#### 3.2.1 HV

In healthy volunteers, all FDR-significant Stage 1 tests involved direct effects (Supplementary Table S3). Five taxa showed a significant direct effect (ADE FDR < 0.05) on at least one SCFA under both methods (ANCOM-BC2 and CLR).

Separately, 16 taxa showed a nominally significant indirect effect (ACME *p* < 0.05) in Stage 1 and were evaluated in Stage 2 (Supplementary Table S4). Of the 16, only one taxon, *Faecalibacterium prausnitzii* (mean relative abundance 2.91%, prevalence 100% in HV), showed a potential Stage 2 indirect effect (bootstrap *p* < 0.05 and permutation *p* < 0.05, under both ANCOM-BC2 and CLR), mediating butyrate through total colonic transit. Specifically, under ANCOM-BC2, higher *F. prausnitzii* abundance was associated with faster transit (a-path = −0.67, *p* = 0.005), and faster transit with higher butyrate (b-path = −0.73, *p* = 0.003), together yielding a significant positive indirect effect of this taxon on butyrate (indirect = 0.49, 95% CI 0.06–0.89, *p* = 0.023); the direct effect of the taxon on butyrate was not significant (*c'* = −0.24, *p* = 0.41), nor was its total effect (*p* = 0.34). Indirect effects on acetate and propionate followed the same direction and comparable magnitude (0.54 and 0.56) but did not reach significance (*p* = 0.097 and 0.098). CLR reproduced this result closely: *a* = −0.65 (*p* = 0.006), indirect effect on butyrate = 0.50 (*p* = 0.022), with acetate and propionate again directionally consistent but non-significant (*p* = 0.073 and 0.070). The butyrate indirect effect also passed both consistency diagnostics: no sign changes across 17 leave-one-out refits under either method, and permutation-test empirical *p* = 0.005 (ANCOM-BC2) and *p* = 0.004 (CLR) (Figure 1). To examine whether this finding was driven by a small subset of participants, we recalculated the taxon-transit and transit-butyrate correlations after excluding each of the 17 participants in turn (Supplementary Figure S2). Both correlations remained negative and showed similar magnitudes across all exclusions, indicating that neither association was driven by any single participant. In addition, *F. prausnitzii* was present in all 17 HV participants (100% prevalence), so this association reflects how much of the taxon each participant carried, not whether they carried it (Supplementary Figure S3, Supplementary Table S5).

**Figure 1:**
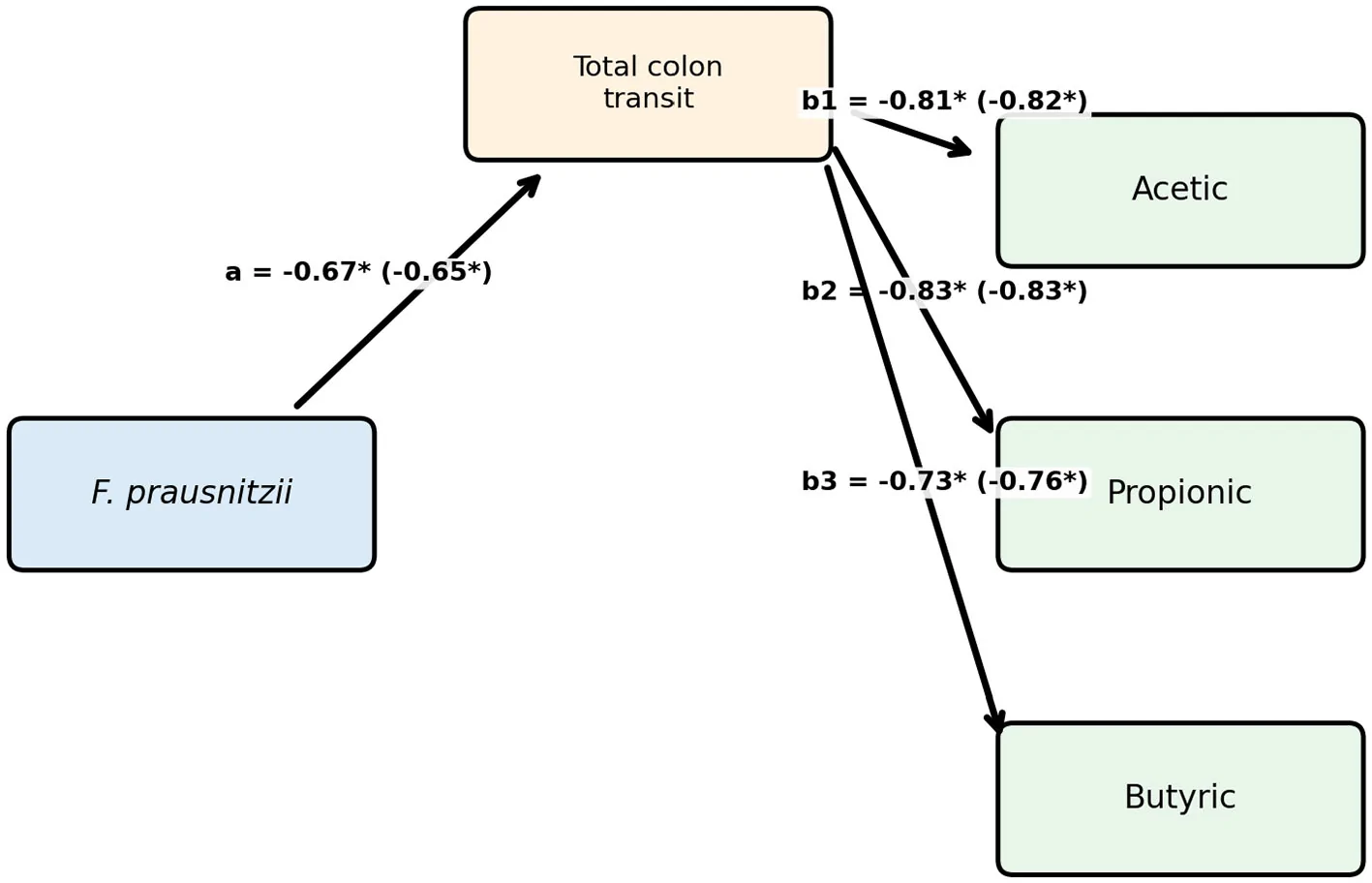
Joint mediation model for *F. prausnitzii* in HV, showing indirect-only mediation of butyrate through total colonic transit. Each path label gives the ANCOM-BC2 estimate followed, in parentheses, by the CLR estimate; asterisks indicate p < 0.05. Point estimates and 95% bootstrap confidence intervals for all paths are provided in Supplementary Table S4.

#### 3.2.2 IBS-C

In IBS-C, two taxa reached FDR < 0.05 for a direct effect under both methods (Supplementary Table S6), one of which (*Bacteroides thetaiotaomicron*) also had a significant indirect effect. Separately, 19 taxa showed a nominally significant indirect effect (ACME *p* < 0.05) and were evaluated in Stage 2 (Supplementary Table S4). Of these 19, two reached the Stage 2 consistency standard: *B. thetaiotaomicron* and *Prevotella* sp. CAG:1031.

*B. thetaiotaomicron* (mean relative abundance 2.04%, prevalence 73.3% in IBS-C) showed a potential Stage 2 indirect effect on all three SCFAs through left colon transit, accompanied by a significant direct effect on all three SCFAs that acted in the opposite direction (a suppressor pattern). Specifically, under ANCOM-BC2, higher *B. thetaiotaomicron* abundance was associated with longer left colon transit (a-path = 0.70, *p* < 0.001), and longer transit with higher levels of all three SCFAs (b-paths = 0.93–1.06, *p* < 0.001 for acetate and propionate, *p* = 0.024 for butyrate), together yielding significant positive indirect effects on acetate (indirect = 0.74, 95% CI 0.27–1.40, *p* = 0.008), propionate (indirect = 0.73, 95% CI 0.35–1.27, *p* = 0.002), and butyrate (indirect = 0.65, 95% CI 0.09–1.35, *p* = 0.039). At the same time, the taxon showed a significant negative direct effect on all three SCFAs (c': acetate −0.96, *p* < 0.001; propionate −0.84, *p* = 0.015; butyrate −0.82, *p* = 0.008), opposing the indirect pathway; because the two pathways act in opposite directions, the total effect was non-significant for all three SCFAs (all *p* > 0.43).

CLR reproduced this result closely: *a* = 0.75 (*p* < 0.001), b-paths 0.95–1.11 (all *p* < 0.05), indirect effects for acetate (0.83, *p* = 0.008), propionate (0.80, *p* = 0.004), and butyrate (0.71, *p* = 0.051), direct effects c' negative for all three SCFAs (−0.98, −0.83, −0.80; *p* < 0.03), and non-significant total effects (all *p* > 0.60): the suppressor pattern, its magnitude, its direction, and the mediating transit segment were consistent across both independent normalization methods for all three SCFAs (Figure 2). All three indirect effects also passed both consistency diagnostics: no sign changes across 15 leave-one-out refits under either method for any of the three SCFAs, and permutation-test empirical *p* = 0.0005 (acetate and propionate, both methods), *p* = 0.0015 (butyrate, ANCOM-BC2), and *p* = 0.0005 (butyrate, CLR). This is the one exception to our general consistency criterion: even though its CLR bootstrap *p*-value for butyrate (0.051) narrowly exceeds 0.05, we retained it because it is significant by the assumption-free permutation test and stable under leave-one-out analysis, under both normalization methods. As a further sensitivity check, we recalculated the a-path and b-path correlations restricted to the 11 taxon-positive samples in which *B. thetaiotaomicron* was detected (prevalence 73.3% in IBS-C); both remained significant (detected-only a-path *r* = 0.78, *p* = 0.005; b-path *r* = 0.67, *p* = 0.025), so this association reflects how much of the taxon participants carried, not just whether they carried it (Supplementary Figure S3, Supplementary Table S5).

**Figure 2:**
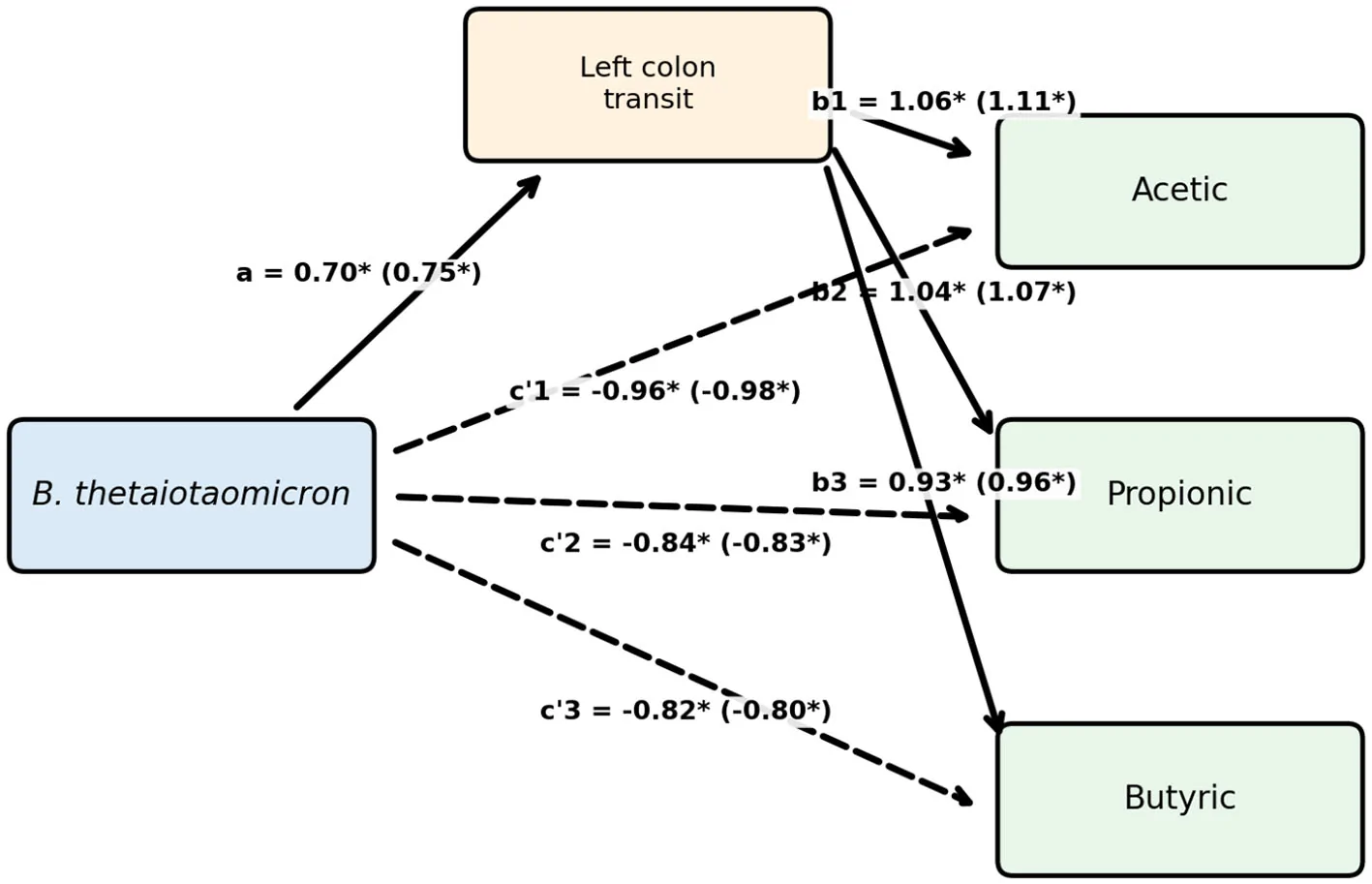
Joint mediation model for *B. thetaiotaomicron* in IBS-C, showing a suppressor pattern (opposing indirect and direct effects) on acetate, propionate, and butyrate through left colon transit. Each path label gives the ANCOM-BC2 estimate followed, in parentheses, by the CLR estimate; asterisks indicate *p* < 0.05. Point estimates and 95% bootstrap confidence intervals for all paths are provided in Supplementary Table S4.

*Prevotella* sp. CAG:1031 showed a potential Stage 2 indirect effect on butyrate through right colon transit (bootstrap *p* < 0.05 and permutation *p* < 0.05, under both ANCOM-BC2 and CLR; a-path = 0.68, *p* = 0.004; indirect effect on butyrate = −0.49, *p* = 0.042; CLR reproduced this closely, indirect = −0.49, *p* = 0.024). However, this taxon was present at a mean relative abundance of only 0.0004% in IBS-C and was detected in just 4 of 15 IBS-C samples; given this extremely low and sparse abundance, we do not count it as a biological meaningful finding.

#### 3.2.3 IBS-D

Similar to HV, in IBS-D, all FDR-significant Stage 1 tests involved direct effects (Supplementary Table S7). Eight taxa showed a significant direct effect (ADE FDR < 0.05) on at least one SCFA under both methods (ANCOM-BC2 and CLR). Separately, 20 taxa showed a nominally significant indirect effect (ACME *p* < 0.05) and were evaluated in Stage 2 (Supplementary Table S4). Of these 20, five reached the Stage 2 selection criteria (Supplementary Table S8): *Bacteroides dorei, Coprococcus comes, Parasutterella excrementihominis, Phascolarctobacterium succinatutens*, and *Prevotella copri*; three of the 20, including *P. succinatutens*, also had a significant direct effect.

*B. dorei* (mean relative abundance 1.76%, prevalence 87.5% in IBS-D) showed a potential Stage 2 indirect effect on propionate through total colonic transit. Under ANCOM-BC2, higher *B. dorei* abundance was associated with longer transit (a-path = 0.51, *p* = 0.010), and longer transit with lower propionate (b-path = −0.49, *p* = 0.089), together yielding a significant indirect effect [indirect = −0.25, 95% CI (−0.58, −0.06), *p* = 0.045]; CLR reproduced this closely (*a* = 0.54, *p* = 0.006; indirect = −0.29, *p* = 0.033). The indirect effect also passed both consistency diagnostics: no sign changes across 24 leave-one-out refits under either method, and permutation-test empirical *p* = 0.0095 (ANCOM-BC2) and *p* = 0.0025 (CLR). As a further sensitivity analysis, we recalculated the a-path and b-path correlations using only the 21 taxon-positive samples in which *B. dorei* was detected (prevalence, 87.5% in IBS-D). The a-path remained significant (*r* = 0.52, *p* = 0.016), whereas the b-path did not reach statistical significance (*r* = −0.38, *p* = 0.087; Supplementary Table S5). Thus, the association between *B. dorei* abundance and transit remained supported in the detected-only subset, but the association between transit and propionate did not. We therefore cannot exclude the possibility that the observed propionate association is partly related to whether *B. dorei* was detected rather than to variation in its abundance among taxon-positive participants. For this reason, *B. dorei* is not included among the main findings of the study, although its Stage 2 results are retained in Supplementary Table S8 for transparency.

*C. comes* (mean relative abundance 0.23%, prevalence 50.0% in IBS-D) showed a potential Stage 2 indirect effect on acetate through right colon transit. Under ANCOM-BC2, higher *C. comes* abundance was associated with shorter transit (a-path = −0.57, *p* = 0.003), and shorter transit with higher acetate (b-path = −0.58, *p* = 0.004), together yielding a significant indirect effect [indirect = 0.33, 95% CI (0.04, 0.60), *p* = 0.024]; CLR reproduced this closely (*a* = −0.58, *p* = 0.001; indirect = 0.33, *p* = 0.022). The indirect effect also passed both consistency diagnostics: no sign changes across 24 leave-one-out refits under either method, and permutation-test empirical *p* = 0.0035 under both methods. As a further sensitivity analysis, we recalculated the a-path and b-path correlations using only the 12 taxon-positive samples in which *C. comes* was detected (prevalence, 50.0% in IBS-D). Neither the a-path (*r* = −0.46, *p* = 0.129) nor the b-path (*r* = −0.50, *p* = 0.099) reached statistical significance, although both point estimates remained similar in magnitude to those obtained from the full sample (Supplementary Table S5). Because restricting the analysis to taxon-positive samples reduced the sample size by half, it is difficult to determine whether the associations were truly weakened or whether the loss of significance mainly reflects reduced statistical power. For this reason, *C. comes* is not included among the main findings of the study, although its Stage 2 results are retained in Supplementary Table S8 for transparency.

*P. excrementihominis* (mean relative abundance 0.30%, prevalence 75.0% in IBS-D) showed a potential Stage 2 indirect effect on acetate through right colon transit. Under ANCOM-BC2, higher *P. excrementihominis* abundance was associated with longer transit (a-path = 0.46, *p* = 0.006), and longer transit with lower acetate (b-path = −0.54, *p* = 0.002), together yielding a significant indirect effect [indirect = −0.25, 95% CI (−0.48, −0.06), *p* = 0.023]; CLR reproduced this closely (*a* = 0.44, *p* = 0.013; indirect = −0.24, *p* = 0.031). The indirect effect also passed both consistency diagnostics: no sign changes across 24 leave-one-out refits under either method, and permutation-test empirical *p* = 0.0080 (ANCOM-BC2) and *p* = 0.0105 (CLR). As a further sensitivity analysis, we recalculated the a-path and b-path correlations using only the 18 taxon-positive samples in which *P. excrementihominis* was detected (prevalence, 75.0% in IBS-D). The a-path was close to null (*r* = 0.04, *p* = 0.862), and the b-path also did not reach statistical significance (*r* = −0.40, *p* = 0.104; Supplementary Table S5). Because the taxon-transit association was no longer evident when the analysis was restricted to detected samples, the full-sample finding may reflect mainly a difference between participants with and without detectable *P. excrementihominis*, rather than a graded association with its abundance among taxon-positive participants. For this reason, *P. excrementihominis* is not included among the main findings of the study, although its Stage 2 results are retained in Supplementary Table S8 for transparency.

*P. copri* (mean relative abundance 4.32%, prevalence 70.8% in IBS-D) showed a potential Stage 2 indirect effect on acetate through total colonic transit. Under ANCOM-BC2, higher *P. copri* abundance was associated with shorter transit (a-path = −0.52, *p* = 0.011), and shorter transit with higher acetate (b-path = −0.57, *p* = 0.016), together yielding a significant indirect effect [indirect = 0.29, 95% CI (0.07, 0.62), *p* = 0.032]; CLR reproduced this closely (*a* = −0.46, *p* = 0.021; indirect = 0.27, *p* = 0.031). The indirect effect also passed both consistency diagnostics: no sign changes across 24 leave-one-out refits under either method, and permutation-test empirical *p* = 0.004 (ANCOM-BC2) and *p* = 0.0095 (CLR). As a further sensitivity analysis, we recalculated the a-path and b-path correlations using only the 17 taxon-positive samples in which *P. copri* was detected (prevalence, 70.8% in IBS-D). Neither the a-path (*r* = −0.25, *p* = 0.333) nor the b-path (*r* = −0.17, *p* = 0.522) reached statistical significance in this subset (Supplementary Table S5). Thus, we could not confirm that this association reflected variation in the abundance of *P. copri* among carriers rather than mainly the difference between participants with and without detectable *P. copri*. For this reason, *P. copri* is not included among the main findings of the study, although its Stage 2 results remain available in Supplementary Table S8 for transparency.

*P. succinatutens* (mean relative abundance 0.42%, prevalence 25.0% in IBS-D), an asaccharolytic, succinate-utilizing, propionate-producing organism, was the only IBS-D taxon to show a reinforcing rather than indirect-only pattern, with potential Stage 2 indirect effects on acetate and propionate through total colonic transit, reinforced by a significant direct effect in the same direction. Specifically, under ANCOM-BC2, higher *P. succinatutens* abundance was associated with shorter transit (a-path = −0.32, *p* = 0.008), and shorter transit with higher acetate (b-path = −0.64, *p* = 0.012) and propionate (b-path = −0.51, *p* = 0.070), together yielding indirect effects on acetate [indirect = 0.21, 95% CI (0.07, 0.40), *p* = 0.013] and propionate [indirect = 0.16, 95% CI (0.06, 0.36), *p* = 0.025]; the indirect effect on butyrate reached nominal bootstrap significance (indirect = 0.12, *p* = 0.046); permutation-test empirical p was 0.060 (ANCOM-BC2) and 0.050 (CLR). The taxon also showed a significant direct effect on acetate in the same direction as the indirect pathway (*c'* = −0.57, *p* < 0.001), so the two pathways reinforced (Figure 3A), yielding a significant total effect on acetate (total = −0.37, *p* = 0.020). CLR reproduced this pattern closely: *a* = −0.33 (*p* = 0.005), indirect effects for acetate (0.22, *p* = 0.006) and propionate (0.17, *p* = 0.015), direct effect on acetate (*c'* = −0.58, *p* < 0.001), and total effect on acetate (total = −0.36, *p* = 0.019). The acetate and propionate indirect effects also passed both consistency diagnostics: no sign changes across 24 leave-one-out refits under either method, and permutation-test empirical *p* = 0.0285 (ANCOM-BC2) and *p* = 0.022 (CLR) for acetate, *p* = 0.0445 (ANCOM-BC2) and *p* = 0.040 (CLR) for propionate.

**Figure 3:**
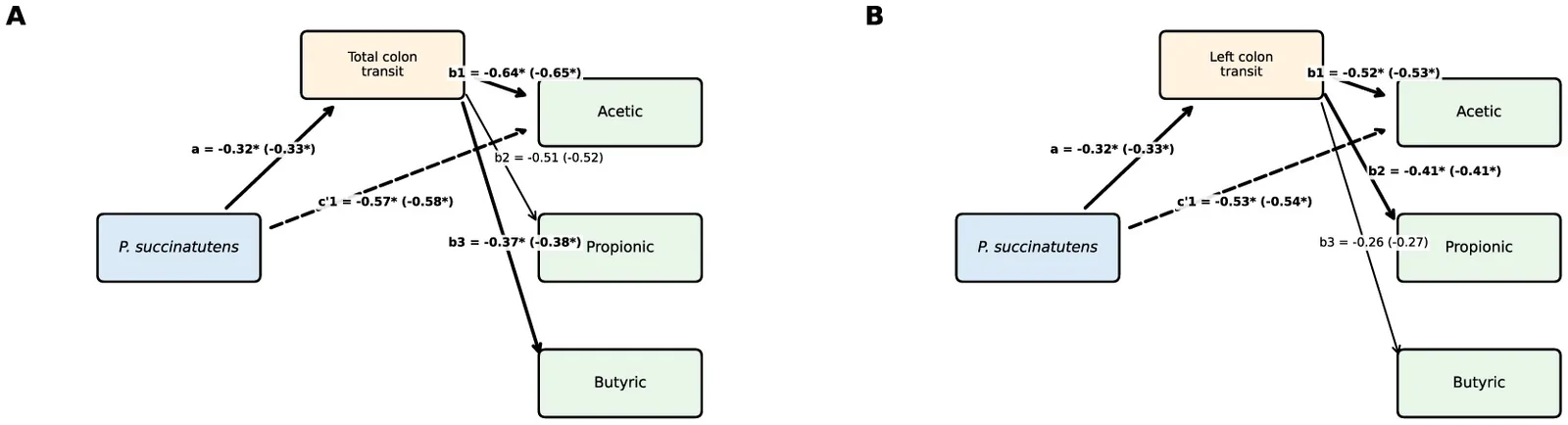
Joint mediation models for *Phascolarctobacterium succinatutens* (IBS-D), the only IBS-D taxon with a potential indirect effect retained after the detected-only sensitivity check. **(A)** A reinforcing pattern on acetate and propionate through total colonic transit. **(B)** The same reinforcing pattern on acetate reproduced independently through left colon transit. Point estimates and 95% bootstrap confidence intervals for all paths are provided in Supplementary Table S4.

*P. succinatutens* also qualified for Stage 2 through left colon transit and showed the same reinforcing pattern on acetate. Under ANCOM-BC2, higher abundance was again associated with shorter transit (a-path = −0.32, *p* = 0.006), with a significant direct effect on acetate in the same direction as the indirect pathway (*c'* = −0.53, p = 0.002) and a significant indirect effect on acetate [0.17, 95% CI (0.04, 0.34), *p* = 0.027], yielding a significant total effect (total = −0.37, *p* = 0.020); the indirect effect on propionate reached nominal bootstrap significance (0.13, *p* = 0.038) but did not meet the selection criteria, and the indirect effect on butyrate was not significant under either method. CLR reproduced this closely: *a* = −0.33 (*p* = 0.005), *c'* = −0.54 (*p* = 0.002), indirect effect on acetate (0.17, *p* = 0.019). The acetate indirect effect also passed both consistency diagnostics: no sign changes across 24 leave-one-out refits under either method, and permutation-test empirical *p* = 0.032 (ANCOM-BC2) and *p* = 0.029 (CLR) (Figure 3B). As a further sensitivity check, we recalculated the a-path and b-path correlations restricted to the 6 taxon-positive samples in which *P. succinatutens* was detected (prevalence 25.0% in IBS-D). Both the a-path (*r* = −0.88, *p* = 0.020) and b-path (*r* = −0.89, *p* = 0.017) remained significant despite the small detected-sample size, so this association reflects variation in how much of the taxon participants carried, not just whether they carried it (Supplementary Figure S3, Supplementary Table S5).

### 3.3 Confounder-adjusted sensitivity analysis

As a sensitivity analysis, we further adjusted both the mediator and outcome models for age, sex, and BMI. After adjustment, only the indirect effects involving *B. thetaiotaomicron* remained significant, including 2 of its 3 taxon-segment-SCFA findings. In contrast, the indirect effects involving *F. prausnitzii* and *P. succinatutens* did not remain significant for any SCFA or transit segment. This loss of statistical significance may mainly reflect reduced statistical power after adding three additional covariates to the already small per-group samples (Supplementary Table S9), rather than necessarily indicating that the underlying associations were substantially changed. However, the present data cannot distinguish these possibilities with confidence.

## 4 Discussion

To our knowledge, this is the first study to formally examine whether colonic transit time statistically mediates the association between gut bacterial abundance and excreted stool SCFA levels in IBS. A significant indirect effect indicates that bacterial abundance is statistically associated with transit time, which is in turn statistically associated with SCFA levels. This interpretation is based on the causal structure specified *a priori* in our directed acyclic graph (Supplementary Figure S4). Although the analysis itself identifies statistical associations and does not establish biological causality, the biological rationale for the mediation analysis is that gut bacteria may influence intestinal transit through the production of neuroactive metabolites, interactions with enteric neurons, or effects on mucosal serotonin signaling (11), while transit time may in turn influence substrate contact time, fermentation duration, and SCFA absorption. In contrast, a significant direct effect indicates that the association between a taxon and SCFA is not explained by the modeled transit pathway and may be consistent with other mechanisms, such as direct metabolite production or substrate utilization.

We conducted the analysis separately within each clinical group and required consistency across two methodologically distinct taxon-abundance normalization approaches (ANCOM-BC2 bias correction and CLR transformation), together with leave-one-out stability, permutation-test support, and an additional detected-only sensitivity analysis in which both the taxon-transit (a-path) and transit-SCFA (b-path) correlations were required to remain significant when restricted to taxon-positive samples. Using these criteria, we identified 7 potential transit-mediated bacteria-SCFA associations involving 3 taxa, with one taxon identified in each of HV, IBS-C, and IBS-D.

Of the three taxa, *F. prausnitzii* (HV) showed the transit-mediated pathway as the only detectable association with SCFA, with no significant direct effect once transit was accounted for (Figure 1), consistent with a statistical pathway in which the taxon-SCFA association operates through transit rather than through a detectable direct effect. Although *F. prausnitzii* is a well-established butyrate producer via the butyryl-CoA:acetate CoA-transferase pathway (21) and its abundance correlates strongly with fecal butyrate in healthy adults (22), our finding of an indirect-only effect suggests that transit may account for the relationship between *F. prausnitzii* and excreted butyrate.

Two taxa departed from this pattern by also showing a significant direct effect. *B. thetaiotaomicron* in IBS-C showed a suppressor pattern, with the indirect and direct pathways acting in opposite directions and canceling in the total association (Figure 2). This finding exemplifies how a single taxon may exert opposing effects on the gut metabolome through distinct mechanistic pathways: one mediated by effects on host physiology (transit) and the other reflecting its metabolic role as a primary degrader of fermentable substrates without a proportional contribution to excreted SCFA. The net cancellation of these effects may explain why simple correlational analyses often fail to detect meaningful microbe-metabolite relationships. In contrast, *P. succinatutens* in IBS-D showed a reinforcing pattern instead, with the two pathways acting in the same direction on acetate through both total and left colon transit (Figures 3A, B), although total and left colon transit are highly correlated (*r* = 0.89, *p* < 0.001). Future studies are required to understand the mechanisms underlying these patterns. For instance, somewhat counterintuitively, slower left colon transit associated with higher *B. thetaiotaomicron* abundance was associated with increased rather than decreased SCFA levels. However, this observation alone does not explain the underlying biological mechanism. One possible explanation, which was not tested in the present study, is that *B. thetaiotaomicron*-associated slowing of transit may prolong substrate exposure and thereby promote fermentation by the broader microbial community, even if *B. thetaiotaomicron* itself does not directly contribute to excreted SCFA. Further studies will be needed to directly test this hypothesis.

In addition, although transit times were not significantly different between the IBS subtypes, except for the borderline difference in left colon transit (*p* = 0.07), the absence of a significant between-group difference in mean transit time does not exclude the presence of a significant within-group taxon-transit association: the between-group comparison examines whether the groups differ in their average transit time, whereas the within-group analysis examines whether variation in the abundance of a specific taxon is associated with variation in transit time among individuals within the same group. Therefore, two groups may have similar mean transit times while still showing different taxon-specific associations with transit within each group.

Several limitations should be acknowledged. First, this study is a secondary, *post-hoc* analysis of a previously published cohort (12) and was not designed *a priori* to test the transit-mediation hypothesis. The relatively small sample size in each clinical group (15–24 participants) limits statistical power, and the findings reported here should therefore be considered exploratory. Second, because this is a cross-sectional study, mediation analysis cannot establish causal relationships. Longitudinal or interventional studies will be needed to examine causality, and reverse or bidirectional relationships, such as effects of transit on microbial composition or effects of SCFA on host physiology, cannot be excluded. Third, the leave-one-out, permutation, and cross-method analyses used to evaluate the internal consistency of the findings were all performed within the same 56 participants. Validation in an independently collected cohort will therefore be necessary to establish external reproducibility. Fourth, medication use and stool characteristics were not available as covariates and remain possible sources of unmeasured confounding. In addition, the limited sample size restricted the number of covariates, including age, sex, and BMI, that could be adjusted for without further reducing statistical power. Fifth, although none of the three reported taxa was identified in more than one clinical group, the present sample size was not sufficient to formally determine whether this pattern reflects true group-specific microbial drivers. Sixth, neither ANCOM-BC2 nor CLR can provide true absolute taxon abundance. Future studies using absolute abundance measurements, such as qPCR, will be important to determine whether the observed mediation associations can be reproduced using absolute bacterial abundance. Finally, transit was measured separately for the right colon, transverse colon, and left colon, whereas stool microbial composition represents an aggregated pool of distal luminal contents and cannot fully capture region-specific microbial ecology or function in the small intestine, at the mucosal surface, or within individual colonic segments (23–27). Therefore, the present data cannot determine whether a mediation association involving a given taxon reflects a truly localized, segment-specific microbial effect, or a whole community effect on segment-specific transit that is indirectly reflected by the overall stool abundance of that microbe.

## 5 Conclusions

Colonic transit may statistically mediate the association between specific gut bacteria and SCFA levels in both health and IBS, suggesting gut motility as one pathway connecting the microbiome to SCFA output, alongside bacteria's direct metabolic effects. These findings are exploratory and reflect statistical association rather than established causality; independent validation in a larger cohort will be needed before their biological or clinical significance can be established.

## Data Availability

Data analyzed for this study were provided as the Supplementary Tables. Analysis codes are provided in a public GitHub repository: https://github.com/qunfengdong/IBS-colonic-transit-SCFA-mediation.

## Supplementary material

Supplementary Figure S1 Flow of taxa through the analysis pipeline, from the initial candidate set to the final potential findings. After defining the shared set of 94 taxa retained by the 10% prevalence filter in ANCOM-BC2 and matched in CLR, direct effects (ADE) and indirect effects (ACME) were evaluated as two parallel analytical branches rather than as sequential filtering steps. In the ADE branch, taxa showing a significant direct effect on at least one SCFA were identified using Benjamini-Hochberg FDR correction (FDR < 0.05) and are reported separately in Supplementary Tables S3, S6, S7: 5 taxa in HV, 2 in IBS-C, and 8 in IBS-D. In the ACME branch, a nominal Stage 1 threshold of *p* < 0.05 was used to nominate candidate taxa for Stage 2 path modeling, resulting in 16 candidates in HV, 19 in IBS-C, and 20 in IBS-D. Among these candidates, the following were retained as potential Stage 2 findings based on bootstrap and permutation significance under both ANCOM-BC2 and CLR, together with leave-one-out stability: 1 in HV, 2 in IBS-C, and 5 in IBS-D. The two IBS-C findings include *Prevotella* sp. CAG:1031, which met the same analytical criteria as the other findings but was excluded because of its extremely low relative abundance in IBS-C (mean 0.0004%; detected in 4 of 15 samples). Of the remaining 7 candidates, a further detected-only sensitivity check, requiring both the a-path and b-path to remain significant (*p* < 0.05) within taxon-positive samples only, excluded four IBS-D taxa (*B. dorei, C. comes, P. excrementihominis, P. copri*), leaving 3 taxa reported as the study's main findings: *F. prausnitzii* (HV), *B. thetaiotaomicron* (IBS-C), and *P. succinatutens* (IBS-D).

Supplementary Figure S2 *Faecalibacterium prausnitzii*, colonic transit, and butyrate in HV (*n* = 17) under two independent abundance-normalization methods. Top row **(A–C)**: ANCOM-BC2 bias-corrected log-abundance. Bottom row **(D–F)**: CLR-transformed abundance. **(A, D)** *F. prausnitzii* abundance vs. total colonic transit (a-path). **(B, E)** Total colonic transit vs. butyrate (b-path). **(C, F)** *F. prausnitzii* abundance vs. butyrate, representing the unadjusted association corresponding to the non-significant direct effect (*c', p* = 0.41). Each panel reports the Pearson correlation and its leave-one-out (LOO) range across all 17 possible single-participant exclusions. The direct/unadjusted association **(C, F)** is nominally positive, whereas the a-path and b-path **(A–B, D–E)** are negative; this sign pattern is expected from the product of two negative path coefficients and does not represent an inconsistency.

Supplementary Figure S3 Relative-abundance distributions for the three reported taxa, restricted to the clinical group in which each was found. Bars show rank-ordered relative abundance (log scale) across all participants in the taxon's reported group; gray bars indicate samples with zero (undetected) abundance. Prevalence (proportion of samples with relative abundance > 0) is reported above each panel.

Supplementary Figure S4 Assumed causal structure (directed acyclic graph) underlying the mediation analysis, showing the taxon-transit-SCFA mediation triangle (a, b, and c′ paths), the measured covariates (age, sex, BMI) adjusted in the sensitivity analysis in Supplementary Table S9, diet (controlled by standardized protocol design rather than statistical adjustment), the unmeasured potential confounders medication use and stool characteristics, and the sequential ignorability identification assumptions stated in full in the figure.

Supplementary Table S1 Input data for the 56-participant analytic cohort: raw MetaPhlAn3 read counts for the 94 analyzed taxa (Raw_Read_Counts), CLR-transformed taxon abundances (CLR_Abundance), ANCOM-BC2 bias-corrected taxon log-abundances (ANCOM-BC2_Log_Abundance), segmental colonic transit times (Colonic_Transit_Time), participant demographics (Demographics), and stool short-chain fatty acid concentrations (SCFA). The three taxon-abundance sheets are taxon-by-sample matrices (rows = participants, identified by Study.ID; columns = taxa); the Colonic_Transit_Time, Demographics, and SCFA sheets give one row per participant with each measured variable as a column; the Demographics sheet reports each participant's age (years), sex, BMI (kg/m^2^), race, and ethnicity (two participants with unrecorded race/ethnicity are left blank). All sheets include a Group column indicating clinical group (HV, IBS-C, IBS-D).

Supplementary Table S2 ANCOM-BC2 bias-corrected log-abundance estimates from a within-group sensitivity analysis (ANCOM-BC2 applied separately within each clinical group), compared with the pooled-cohort estimates used throughout this manuscript for all 94 taxa.

Supplementary Table S3 Significant Stage 1 mediation results in HV (ADE FDR < 0.05). ACME_est, ADE_est, and Total_est are the point estimates of the average causal mediation effect (indirect, transit-mediated effect), average direct effect (transit-independent effect), and total effect (ACME + ADE, equivalent to the taxon's unadjusted association with the SCFA), respectively.

Supplementary Table S4 Full Stage 2 path model parameter estimates for all taxa and groups, including all taxa evaluated in Stage 2 regardless of consistency status. Each row is one estimated model parameter, identified by lhs, op, and rhs (left-hand side, operator, and right-hand side of the corresponding lavaan model syntax): op = “~” denotes a regression path (an effect of rhs on lhs), “~~” a variance or covariance term, and “:=” a quantity computed from other paths (an indirect or total effect). The label column gives the path's short name as used in the manuscript text and Figures 1–3: a is the taxon-to-transit path; b1, b2, and b3 are the transit-to-acetate, -propionate, and -butyrate paths, respectively; c1, c2, and c3 are the corresponding direct taxon-to-SCFA paths; ind_acetic, ind_propionic, and ind_butyric are the computed indirect effects (a × the corresponding b); and total_acetic, total_propionic, and total_butyric are the computed total effects (indirect + corresponding c). Rows with op = “~~” and lhs = rhs = “Taxon” report the fixed sample variance of the z-scored taxon abundance variable.

Supplementary Table S5 Prevalence (proportion of samples with relative abundance >0) and detected-only (taxon-positive-only) taxon-transit (a-path) and transit-SCFA (b-path) Pearson correlations for the three reported taxa and for the four additional IBS-D taxa (*Bacteroides dorei, Coprococcus comes, Parasutterella excrementihominis*, and *Prevotella copri*) that met the Stage 2 selection criteria but were excluded from the reported findings because they did not meet this criterion.

Supplementary Table S6 Significant Stage 1 mediation results in IBS-C (ADE FDR < 0.05). The column structure is the same as Supplementary Table S3.

Supplementary Table S7 Significant Stage 1 mediation results in IBS-D (ADE FDR < 0.05). The column structure is the same as Supplementary Table S3.

Supplementary Table S8 Significant indirect effects from Stage 2 path models (*p* < 0.05). Five rows, *Prevotella* sp. CAG:1031 (IBS-C), *Bacteroides dorei, Coprococcus comes, Parasutterella excrementihominis*, and *Prevotella copri* (all IBS-D), met the same Stage 2 selection criteria as the other rows but are excluded from the study's main findings: *Prevotella* sp. CAG:1031 because of its extremely low relative abundance (0.0004% mean in IBS-C, detected in 4 of 15 samples), and the four IBS-D taxa because their Stage 2 indirect effects did not meet the additional detected-only sensitivity criterion (Supplementary Table S5).

Supplementary Table S9 Unadjusted vs. age-, sex-, and BMI-adjusted average causal mediation effect (ACME) estimates for the 7 taxon-segment-SCFA indirect effects reported in this study, from the confounder-adjusted sensitivity analysis.
